# Predicting success in medical school: a longitudinal study of common Australian student selection tools

**DOI:** 10.1186/s12909-016-0692-3

**Published:** 2016-07-22

**Authors:** Ruth M. Sladek, Malcolm J. Bond, Linda K. Frost, Kirsty N. Prior

**Affiliations:** School of Medicine, Flinders University, Adelaide, Australia; Prideaux Centre for Research in Health Professions Education, School of Medicine, Flinders University, GPO Box 2100, Adelaide, 5001 Australia

**Keywords:** School admissions criteria, Medical students, Interviews, Predictive validity, Australia

## Abstract

**Background:**

Medical student selection and assessment share an underlying high stakes context with the need for valid and reliable tools. This study examined the predictive validity of three tools commonly used in Australia: previous academic performance (Grade Point Average (GPA)), cognitive aptitude (a national admissions test), and non-academic qualities of prospective medical students (interview).

**Methods:**

A four year retrospective cohort study was conducted at Flinders University Australia involving 382 graduate entry medical students first enrolled between 2006 and 2009. The main outcomes were academic and clinical performance measures and an indicator of unimpeded progress across the four years of the course.

**Results:**

A combination of the selection criteria explained between 7.1 and 29.1 % of variance in performance depending on the outcome measure. Weighted GPA consistently predicted performance across all years of the course. The national admissions test was associated with performance in Years 1 and 2 (pre-clinical) and the interview with performance in Years 3 and 4 (clinical). Those students with higher GPAs were more likely to have unimpeded progress across the entire course (OR = 2.29, 95 % CI 1.57, 3.33).

**Conclusions:**

The continued use of multiple selection criteria to graduate entry medical courses is supported, with GPA remaining the single most consistent predictor of performance across all years of the course. The national admissions test is more valuable in the pre-clinical years, and the interview in the clinical years. Future selections research should develop the fledgling research base regarding the predictive validity of the Graduate Australian Medical School Admissions Test (GAMSAT), the algorithms for how individual tools are combined in selection, and further explore the usefulness of the unimpeded progress index.

## Background

Student selection and assessment in medical education share an underlying high stakes context with the need for valid and reliable tools [1, 2]. Yet evidence for the predictive validity of commonly used selection tools in Australian graduate entry medicine is sparse at best. We examine the predictive validity of such tools in a longitudinal study of student performance.

Historically it has been commonplace for medical schools to use several different measures in combination to select students [3]. Studies examining a range of factors associated with success in medical school were first systematically reviewed in 2000 [3]. These included previous academic ability, personality, learning styles, interviews, references, personal statements, sex and ethnicity. With the exception of academic or cognitive performance (previous academic results or Medical College Admissions Test (MCAT) scores) predicting success in undergraduate achievement, few other conclusions were possible due to the lack of research.

By the time of a global Consensus Statement in 2010 [1], medical educationalists concluded that both Grade Point Average (GPA) as a measure of previous academic ability and the Medical College Admissions Test (MCAT) had clear evidence for their predictive capacity, but only within a North American context. For other countries, GPA and newer tests such as the Graduate Australian Medical School Admissions Test (GAMSAT) used in Australia and elsewhere were yet to demonstrate credible support. Only the new methodology of a Multiple Mini Interview (MMI) was noted to have substantial evidence in support of its reliability and predictive validity both within and beyond North America [1].

Selection tools in medical education were again systematically reviewed in 2015 [4]. Findings mirrored in part the earlier findings, with clear support for previous academic performance as a predictor of success. Additionally it was concluded that structured interviews, MMIs, and two more recent selection methods, the Situational Judgement Test (SJT) and Selection Centres, were more effective than other tools. However the evidence for cognitive aptitude tests (such as MCAT, GAMSAT, and others) was mixed, with a lack of definitional boundaries around what is meant by ‘aptitude’ with consequent inherent differences between these tools. This means, for example, that the body of research evidence about MCAT does not necessarily generalise to other tests such as GAMSAT. Therefore, each tool represents an independent test in need of its own empirical support [4].

Student selection into graduate entry medicine in Australia broadly mirrors the approaches summarised by Patterson et al. [4] and as generally used worldwide [3]. While all publicly-funded graduate medical courses in Australia have individualised selection algorithms (i.e., models of how the results of different assessments are weighted and combined), they share the same assessment criteria commonly used elsewhere: prior academic performance (measured using Grade Point Average (GPA) usually for an undergraduate degree); cognitive aptitudes (measured using GAMSAT) and non-academic qualities (measured using a selection interview, except for one university). GPA and selection interviews are tools readily identified in most educational systems, whereas GAMSAT is Australian in origin.

GAMSAT is a cognitive aptitude test designed to assess the capacity to undertake high level intellectual studies, and comprises three sections: reasoning in the humanities and social sciences (Section 1), written communication (Section 2) and reasoning in the biological and physical sciences (Section 3) [5]. This written examination is highly standardised (same test, day, and rules for all applicants) and considered the single most reliable tool used by Australian graduate medical schools. While originally developed solely for medicine, GAMSAT is now used by medical, dental, optometry, podiatric medicine and veterinary medicine courses across Australia, United Kingdom and Ireland [6]. Interest in the predictive validity of GAMSAT is therefore transnational.

Despite the need for a defensible approach to selection and substantial growth in GAMSAT use (by discipline and by countries), there is a surprising dearth of research into (1) the predictive validity of GAMSAT, and (2) the commonly used GAMSAT, GPA and interviews. Only seven papers have examined these tools in the Australian context [7–13] with the most noteworthy being Puddey and Mercer [11]. While findings are reported for a single institution only, their longitudinal study includes all three predictors of GAMSAT, GPA and Interviews, and outcome measures covering all years of the course. This makes it the singularly most comprehensive study relevant to graduate entry medical school selection in Australia [11]. No other studies have used outcomes covering all years of a course [7–10, 12, 13].

When the three selection tools are considered separately, research supports undergraduate GPA as a predictor of performance in graduate entry medicine across a range of indicators [3]. Indeed, Puddey and Mercer [11] found GPA to be the strongest predictor though its strength diminished as the course progressed. GAMSAT arguably offers attractive face validity. However, reports of its predictive utility are conflicting. Puddey and Mercer [11] are the exception, finding that GAMSAT and GPA together predicted performance across the entire course. While they report preliminary evidence of an association between their interview and performance as the course becomes more clinically-oriented in later years, they note the nature of this relationship is yet to be determined given the small sample size. Only three other studies have found some relationship between an interview and academic performance (or in one case, clinical reasoning skills) [7, 8, 10].

### Background to this study

This study is contextualised within an Australian graduate entry medical school environment at Flinders University in South Australia. Flinders was the first university to offer a graduate entry medical course in Australia in 1996 and one of the three universities that commissioned the development of GAMSAT. Its selection tools have remained constant over time and its broad approach has informed other emerging graduate courses.

Given the relative dearth of research relating to student selection into graduate entry medicine in Australia, this study’s aim was to investigate the degree to which the three elements (GAMSAT, GPA and Interview score) of the Flinders selection model predict performance across all four years of its medical course. The study builds on findings from the only other published longitudinal study of all commonly-used selection criteria across all years of a curriculum in an Australian graduate medical school [11]. However, unlike Puddey and Mercer [11], a number of disaggregated outcomes within each year were used rather than an aggregated annual outcome. This decision was taken on the premise that the three selection tools may be differentially predictive of different course components. This may be in terms of either content (e.g., social science vs. medical science vs. clinical skills) or timing (e.g., Year 1 vs. Year 4), or both. Finally, a composite outcome measure was also incorporated and referred to as ‘unimpeded progress’. A potentially important consideration is that GAMSAT was designed originally to select students into the Flinders’ course. If this tool was to predict performance in any context, then these associations would be expected in this cohort particularly.

## Methods

### Participants

Data were collated retrospectively for 382 students from four entry cohorts (2006–2009) of the graduate entry Flinders University medical course. Table 1 describes these cohorts according to age, gender, previous degree category [14], rural origin and state of origin.

**Table 1:** Socio-demographic characteristics of the study cohorts and total sample

	2006 (*N* = 71)	2007 (*N* = 91)	2008 (*N* = 109)	2009 (*N* = 111)	Total (*N* = 382)
Age (mean, SD)	25.4 (6.1)	24.6 (4.0)	25.7 (5.9)	25.6 (6.4)	25.4 (5.7)
Gender (*n*, % female)	37 (52.1)	53 (58.2)	57 (52.3)	62 (55.9)	209 (54.7)
Previous qualification^a, b^ (*n*, %)					
Health Profession	7 (9.9)	14 (15.4)	26 (24.1)	13 (11.8)	60 (15.7)
Biomedical Science	13 (18.3)	28 (30.8)	31 (28.7)	49 (44.5)	121 (31.7)
Other Biology	34 (47.9)	33 (36.3)	35 (32.4)	36 (32.7)	138 (36.1)
Physical Science	9 (12.7)	8 (8.8)	8 (7.4)	7 (6.4)	32 (8.4)
Non-science	8 (11.3)	8 (8.8)	8 (7.4)	5 (4.5)	29 (7.6)
Rural origin^c^ (*n*, %)	15 (22.7)	22 (24.2)	29 (26.6)	31 (27.9)	97 (25.4)
State of origin SA (*n*, %)	47 (66.2)	51 (56.0)	65 (59.6)	62 (55.9)	225 (58.9)
Impeded progress (*n*, %)	26 (36.6)	43 (47.3)	46 (42.2)	47 (42.3)	162 (42.4)

Notes. ^a^In 2008 previous qualification was available for only 108 students

^b^In 2009 previous qualification was available for only 111 students

^c^In 2006 rural origin was available for only 66 students

#### Exclusion criteria

Three student sub-quotas were excluded (international, indigenous and Parallel Rural Community Curriculum students) because each of these entry pathways uses selection criteria not directly comparable with the standard entry procedures.

### Flinders selection criteria

Students require an undergraduate degree with no pre-requisite subjects. An applicant’s GAMSAT total score is the sole basis for an interview offer (unlike other Australian courses), with no minimum GPA mandated. After interview, a ranking score is calculated by equally weighting GAMSAT total score, GPA, and Interview score to determine whether a place is offered. Both GAMSAT and GPA are weighted as described below.

#### GAMSAT

At Flinders an overall score is calculated with Section 3 being double weighted (termed wGAMSAT).

#### Grade point average

A weighted percentage score (wGPA) is calculated to represent academic performance across applicants’ final three years of undergraduate study. It is calculated as [(GPA1 x 1) + (GPA2 x 2) + (GPA3 x 3)]/6 x 100. Although GPA is universally accepted as reflecting the ability to undertake higher education, in the current context applicants’ GPAs reflect prior academic performance across diverse degrees from a variety of tertiary institutions.

#### Interview

wGAMSAT is used to rank applicants for interview. The Flinders interview is semi-structured and conducted by a panel of two to three interviewers. Six domains are evaluated (communication skills, motivation, learning style, decision making, prosocial attitude, personal management) and a global assessment rating is also given. Possible scores range from 0 (‘unacceptable’) to 5 (‘outstanding’). All scores from all interviewers are summed and converted to a percentage.

### Outcome variables

The focus of the course from an educational perspective changes across the four years. In order to examine potentially different predictive relationships across the course, outcomes for each year were used. Only topics for which continuous data were available were included. In Years 1 and 2 performance measures were predominantly academic results (non-clinical), while in Years 3 and 4 clinical performance was also quantified. When a student was required to repeat a topic, initial results were analysed.

#### Years 1 and 2

Years 1 and 2 are generally regarded as ‘pre-clinical’ years as they occur prior to major clinical placements in Years 3 and 4. Percentages were aggregated and averaged according to year, semester and theme (KHI: Knowledge of Health and Illness; DPS: Doctor, Profession and Society; D & P: Doctor and Patient). These three themes are the organising structure for topics, and continue across each year of the course. Generally speaking, KHI is more ‘science’ oriented, DPS has a more ‘social science’ focus, and D & P encompasses clinical skills.

Year 1, Semester 1 KHI comprised Human Homeostasis and Identity, and Microbes and Defence, while Semester 2 was Cardiovascular System and Human Life Cycle. The full year DPS score comprises Health Psychology and Medicine and Culture. In Year 2, Semester 1 KHI comprised only Gastrointestinal System while Semester 2 was Brain and Behaviour.

Categorical indicators of performance were ‘unimpeded progress Years 1 and 2’. This yes/no variable was used to categorise students who had any interruptions during the medical course such as the requirement for supplementary assessment (academic or medical), failing a year, taking a leave of absence (for academic or personal reasons), or withdrawing from the course.

#### Years 3 and 4

Years 3 and 4 are regarded as ‘clinical’ years, with students undertaking major rotations in a range of clinical environments. In each of Years 3 and 4 a percentage mark was obtained to reflect the total year’s performance. In Year 3 the D & P Objective Structured Clinical Examination (OSCE) score was also available. In Year 4 an overall clinical performance score (ITA: ‘in-training assessment’) was calculated, with scores from 0 (‘falls far short of requirements’) to 7 (‘of excellent standard’) summed across five placements and converted to a percentage. A final ranking of students within their cohort, based on aggregate performance across Years 3 and 4 was also recorded. As for Years 1 and 2, a categorical variable was calculated for ‘unimpeded progress Years 3 and 4’. An overall variable termed ‘any unimpeded progress’ (Years 1 through 4) was also derived.

### Statistical analysis

Data were collated and analysed using IBM SPSS (version 22).

For each continuous outcome measure, an analysis of covariance was conducted (ANCOVA) with the effect of student cohort (the factor) first removed before the joint effects of wGAMSAT, wGPA and Interview were considered. Results are presented as partial eta squared coefficients (η^2^_p_) expressed as a percentage. For categorical variables, logistic regression was used, with forced entry of student cohort at step 1, followed by wGAMSAT, wGPA and Interview at step 2. Results are presented as Odds Ratios.

## Results

### Student characteristics

The sociodemographic characteristics of each cohort are reported in Table 1. The mean age was similar (F_(3,378)_ = 0.73, *p* = .532), as was the gender distribution (*χ*^2^_(3)_ = 0.97, *p* = .809). Between 50 and 60 % of each cohort was female. A significant increase in students from a biomedical science background was noted from 2006 to 2009 (*χ*^2^_(3)_ = 14.71, *p* = .002). A steady proportion of students with a rural background was evident (*χ*^2^_(3)_ = 0.75, *p* = .861), as was the proportion of students from the University’s home state (*χ*^2^_(3)_ = 2.32, *p* = .509). The rates of impeded progress across the course were similarly equivalent across cohorts (*χ*^2^_(3)_ = 1.85, *p* = .604).

### Predictor variables (Table 2)

Summary statistics for predictor variables failed to reveal any significant relationship between wGPA and GAMSAT. There was a significant but small negative relationship between the Interview and both wGPA and wGAMSAT, respectively.

**Table 2:** Summary statistics for predictor variables

	Range	Mean	(SD)	1	2
1. wGPA	3.02–7.00	6.24	(0.60)	-	
2. wGAMSAT	50–85	62.84	(5.34)	.10	-
3. Interview	35.20–100.00	73.09	(12.10)	−.12^*^	−.19^***^

Notes. *p < .05, ***p < .001

### Analysis of continuous outcomes (Table 3)

There is an early significant cohort effect in Years 1 and 2 of the course, however this becomes non-significant as the course progresses. In terms of the three predictor variables, consistent relationships can be seen according to the pre-clinical and clinical years (‘Years 1 and 2’, and ‘Years 3 and 4, including Final Course Ranking’, respectively). wGAMSAT predicts pre-clinical performance but little else. Conversely, the Interview predicts clinical but not pre-clinical performance. wGPA predicts both pre-clinical and clinical performance.

**Table 3:** Associations between selection criteria and continuous outcome measures (including cohort effects)

Outcome variable	Cohort	wGAMSAT	wGPA	Interview	F _model_	R^2^_model_
Year 1, Semester 1, KHI	11.3^***^	10.4^***^	14.5^***^	0.3	23.57^***^	27.5
Year 1, Semester 2, KHI	11.2^***^	8.0^***^	16.3^***^	0.0	23.39^***^	27.6
Year 1, DPS (full year)	7.8^***^	1.5^*^	11.1^***^	0.5	13.16^***^	17.5
Year 2, Semester 1, KHI	7.7^***^	2.3^**^	7.7^***^	0.3	11.46^***^	15.8
Year 2, Semester 2, KHI	25.8^***^	4.6^***^	5.2^***^	0.4	24.84^***^	29.1
Year 3, D & P OSCE	1.0	0.9	2.6^**^	7.0^***^	6.51^***^	10.0
Year 3, Total Score	4.2^**^	2.2^**^	7.2^***^	4.5^***^	10.79^***^	15.8
Year 4, ITA Score	0.3	1.0	2.1^**^	3.5^***^	4.41^***^	7.1
Year 4, Total Score	0.8	0.7	5.2^***^	3.0^***^	6.26^***^	9.5
Final Course Ranking	1.9	0.8	8.4^***^	5.8^***^	10.10^***^	14.6

Notes. Effect sizes are partial eta squared coefficients (η^2^_p_) expressed as a percentage

(2 % = small, 13 % = medium, 26 % = large) [19]

*p < .05, **p < .01, ***p < .001

### Analysis of categorical outcomes (Table 4)

Students with a higher wGPA were more likely to have had unimpeded progress across all years of the course (OR = 2.29, Years 1 to 4). While the Interview was not associated with unimpeded progress in any year, students with a higher wGAMSAT were more likely to have had unimpeded progress in the pre-clinical years only (Years 1 and 2).

**Table 4:** Multivariate associations between selection criteria and unimpeded progress (forced entry logistic regression)

	*Cohort*	*wGAMSAT*		*wGPA*		*Interview*			
Period	Wald	OR	[95 % CI]	OR	[95 % CI]	OR	[95 % CI]	*χ* ^2^_model_	R^2^_model_
Years 1 and 2	6.97	1.09^***^	[1.04–1.14]	2.39^***^	[1.63–3.52]	1.01	[0.99–1.03]	38.62^***^	13.2
Years 3 and 4	5.87	1.01	[0.95–1.06]	2.41^***^	[1.60–3.62]	1.01	[0.99–1.03]	26.10^***^	10.5
Years 1 to 4	3.18	1.04	[0.99–1.09]	2.29^***^	[1.57–3.33]	1.00	[0.99–1.02]	26.59^***^	9.0

Notes. Odds Ratios represent effect sizes (1.5 = small, 2.5 = medium, 4.0 = large) [20]

***p < .001

## Discussion

wGPA was the strongest predictor of academic and clinical performance across all four years of the Flinders course. A higher wGPA was also the most useful indicator of unimpeded progress through the course. Consistent with extant research, these findings provide further support for undergraduate wGPA as a critical selection criterion for graduate entry medicine. The observation that each selection tool had some predictive utility, albeit differentially across the course, continues to support the use of multiple selection tools. For example, wGAMSAT was most clearly associated, with performance in Years 1 and 2 (consistent with less clinical, but a more academic focus), and the Interview with performance in Years 3 and 4 (consistent with these years having more clinical focus). While effect sizes were generally small, the role of the three selection criteria was remarkably consistent at the topic level within each year.

These findings make intuitive sense. Most applicants plan and prepare for GAMSAT sometimes years in advance of sitting the test. This type of preparatory learning (from textbooks and courses) focuses largely on the same content domains as Years 1 and 2, with applicants often directed to University-level biology and chemistry books in preparation for GAMSAT. Further, most in-course assessments are by written examination. On the other hand, the interview, which to a large extent measures communication, places greater emphasis on the skills required in learning and practising medicine, and those necessary for in-course assessments (such as the Objectively Structured Clinical Examination) which feature prominently in the clinical years.

Results support the predictive validity of the Flinders Interview. At a time when MMIs are gaining momentum as a replacement for traditional interviews, with the appeal of being “evidence based”, this is an interesting finding. However structured interviews, such as Flinders’, have some research support [4]. Our semi-structured interview is re-written each year using the same underlying framework, and has a strict administration and scoring protocol that is unchanging. It is arguably important that medical schools understand the predictive abilities of local approaches before committing to costly and potentially unnecessary changes in pursuit of an emerging trend. Overall, Flinders’ current selection model predicted between 7.1 and 29.1 % of performance across the course. This is consistent with others’ findings that about 10 % to 30 % of variance is accounted for by the selection criteria under consideration depending on the course and outcomes measured [11]. As similar as these results appear, more variance in performance is left unexplained than explained by these selection tools. Still, given the many other factors that likely impact performance (curriculum, life events etc), even small predictive relationships are arguably noteworthy. As a comparison, in the case of educational interventions, effect sizes of .20 or less with small gains in learning are considered effective [15].

‘Unimpeded progress’ was a composite index so the extent to which individual components were associated with selection criteria is unknown. Interpretation of the demonstrated association between a poorer GPA and progression delays is limited as it included ‘personal leave’ which could reflect struggling in the course, but could also, for example, represent sickness, conference or maternity leave. Yet it is noteworthy that between 36.6 and 47.3 % of students had impeded progress for some reason at some time and the absolute combined student number was sizeable (162/382, 42.2 %, Table 1). Student attrition [16] and academic struggling [17] in medical school, just two of the included components, have both been associated elsewhere with poorer prior academic achievement (e.g., GPA), although Flinders’ attrition rate is typically low (10/382 or 2.6 % for the reported cohorts). Regardless, findings suggest a complexity in medical course delivery (presumably impacting administration and academic management) that is not necessarily recognised in simpler metrics such as student attrition. In this study it was not possible retrospectively to obtain the level of detail necessary to fully understand these findings, although it remains a fertile ground for future prospective research.

A more detailed consideration of the way in which multiple assessments are combined may shed additional light on the importance on this study’s findings. As argued by Patterson et al. [4] it is important to understand how the collective use of selection tools works. While the use of GAMSAT, wGPA and an Interview is nearly ubiquitous in selecting students for graduate entry medicine in Australia, the manner in which these tools are used varies. At Flinders, applicants are first ranked for interview solely using wGAMSAT. Given the current findings, this policy may mean that Flinders is effectively selecting applicants for interview who are more likely to perform in the pre-clinical years, but at the expense of prospective students who might perform more consistently across the whole course, or perform better in later clinical assessments. An alternative proposition could be presented on pedagogical grounds. One foundation of a Problem Based Learning curriculum, as used by Flinders, is that prior knowledge is the basis of new knowledge [18]. If true, success in pre-clinical years may be an important predictor of subsequent performance independent of GAMSAT scores, and therefore preferencing applicants for early success is entirely reasonable. Further disaggregation of GAMSAT Section scores (i.e., as opposed to the GAMSAT Total used in this study) and associations with individual topic results may inform this proposal.

The limitations of selection research are well known. For example, the current research reflects the problems of attenuated range (i.e., only those ‘selected’ into medicine are included in analyses), and year by year variations in both selection tools (e.g., different interview questions every year) and assessments. Additionally, only reasonably small predictive relationships can be expected given the range of factors likely to impact in-course performance. Notwithstanding these limitations, several significant and sizeable relationships were found and it is possible that these are under-estimates due to the statistical artefact of attenuated range.

Data reliability was robust as primary sources were used to verify records. While not all topic assessments could be included (due to some pass/fail assessments including yearly results) this study could examine selected results at a topic level across the entire course. It was possible to see, for example, that wGPA and GAMSAT predicted performance in topics with quite different foci, such as KHI1 (with a more science focus). Aggregated data would have obscured such detail.

Even though this is a single site study, the tools examined are ubiquitous across Australia and are used in other countries. The findings for GAMSAT, which is now used in a growing number of other countries, have particular transnational relevance as they provide empirical evidence regarding its predictive validity in a research landscape which is fledgling. We do not necessarily suggest the research area has been neglected; rather graduate entry medicine is still relatively young in Australia and others countries outside of North America. Australia’s first graduating cohort (from Flinders) was as recent as 1999.

It is the high stakes context that begs further validation research in relation to GAMSAT, and this study provides further incremental evidence in this field [11]. GAMSAT continues to need its own body of research independent of other aptitude tests, given all aptitude tests are not the same and are unlikely to ever be the focus of a comparative study [3]. We echo others’ conclusions regarding the need for more research into the validity of GAMSAT [4, 11].

## Conclusions

In conclusion, different selection tools predict different outcomes throughout a graduate entry medical programme. GPA remains an important performance predictor across the curriculum whereas GAMSAT is predictive in the early (pre-clinical) years, and a semi-structured panel interview is predictive in the later (clinical) years. While findings confirm the value of using multiple tools, the algorithm for how these are used in combination with each other remains a fertile ground for further research. Further longitudinal research is required to build the fledgling research base in relation to the predictive validity of GAMSAT. Finally and more broadly, future research should further explore the usefulness of the impeded progress index as a relevant outcome with possible implications for course administration and academic management.

## Abbreviations

GAMSAT, Graduate Australian Medical Schools Admissions Test; GPA, grade point average; MCAT, Medical College Admissions Test; MMI, multiple mini interview; wGAMSAT, weighted Graduate Australian Medical Schools Admissions Test; wGPA, weighted grade point average
